# How to Prepare a 1 to 1,000 Solution of Corrosive Sublimate

**Published:** 1884-11

**Authors:** 


### How to Prepare a 1 to 1,000 Solution of Corrosive Sublimate.

In view of the extensive use of this agent as an antiseptic, it will be interesting to note that Sir Joseph Lister writes to the *British Medical Journal* (May 24, 1884), that one drachm by weight of a solution of one part of corrosive sublimate in one and a half parts of glycerine contains two-fifths its weight, or twenty-four grains of the sublimate. This, multiplied by 1,000 (the proportion of water required), gives 24,000 grains, which is very nearly three pints. It is, however, much more convenient to use fluid measure, than weight, and a fluid drachm of glycerine solution referred to requires four pints of water to produce the 1 to 1,000 solution.

